# Effects of dietary yeast inclusion and acute stress on post-prandial whole blood profiles of dorsal aorta-cannulated rainbow trout

**DOI:** 10.1007/s10695-016-0297-0

**Published:** 2016-09-27

**Authors:** David Huyben, Aleksandar Vidakovic, Andreas Nyman, Markus Langeland, Torbjörn Lundh, Anders Kiessling

**Affiliations:** Department of Animal Nutrition and Management, Swedish University of Agricultural Sciences, Box 7024, 750 07 Uppsala, Sweden

**Keywords:** Rainbow trout, Yeast, Aquaculture, Dorsal aorta cannulation, Buffering capacity, Anaemia

## Abstract

Yeast is a potential alternative to fish meal in diets for farmed fish, yet replacing more than 50 % of fish meal results in reduced fish growth. In a 4-week experiment, 15 rainbow trout (*Oncorhynchus mykiss*) were cannulated and fed three diets each week: 30 % fish meal as a control (FM); 60 % replacement of fish meal protein, on a digestible basis, with *Saccharomyces cerevisiae* (SC); and 60 % replacement with *Wickerhamomyces anomalus* and *S. cerevisiae* mix (WA). Blood was collected at 0, 3, 6, 12 and 24 h after feeding. In the final week, fish were exposed to a 1-min netting stressor to evaluate possible diet–stress interactions. Significant increases in pH, TCO_2_, HCO_3_ and base excess were found after fish were fed the SC and WA diets compared with FM, which elevated blood alkaline tides. Yeast ingredients had lower buffering capacity and ash content than fish meal, which explained the increase in alkaline tides. In addition, fish fed the WA diet had significantly reduced erythrocyte area and fish fed SC and WA diets had increased mean corpuscular haemoglobin levels, indicating haemolytic anaemia. Higher levels of nucleic acid in yeast-based diets and potentially higher production of reactive oxygen species were suspected of damaging haemoglobin, which require replacement by smaller immature erythrocytes. Acute stress caused the expected rise in cortisol and glucose levels, but no interaction with diet was found. These results show that replacing 60 % of fish meal protein with yeasts can induce haemolytic anaemia in rainbow trout, which may limit yeast inclusion in diets for farmed fish.

## Introduction

The aquaculture industry relies on fish meal and soy as major protein sources in diets for farmed fish; however, food demand and environmental costs have increased the need for ingredients of non-human interest (Tacon and Metian 2008; Naylor et al. 2009; FAO 2014). Yeast is a single cell protein (SCP) produced from fermentation of agricultural by-products and used in biofuel, brewery and baking processes, but rarely used as a protein source in human food (Ravindra 2000). In fish research, only a few studies have replaced soy and fish meal with yeasts, such as *Saccharomyces cerevisiae*, in diets for rainbow trout (*Oncorhynchus mykiss*) (Mahnken et al. 1980; Rumsey et al. 1992; Martin et al. 1993; Hauptman et al. 2014; Sealey et al. 2015). At most, Hauptman et al. (2014) successfully replaced 38 % of fish meal (11 % of diet) with *S. cerevisiae*, but higher inclusion resulted in negative effects on weight gain and feed conversion even when feed intake and protein content were equal between fish meal- and yeast-based diets. More research is needed to explain the cause of reduced performance when fish are fed diets with high yeast inclusion.

Yeast and other SCP contain tenfold higher levels of nucleic acid, mainly from RNA, than meat and plant ingredients (Kihlberg 1972; Jonas et al. 2001). High quantities of nucleic acid cannot be safely metabolised by humans, as they result in high uric acid levels (hyperuricaemia) and gout (Waslien et al. 1970; Fox 1981). On the other hand, fish produce higher levels of uricase than other animals and are thought to be able to metabolise higher levels of nucleic acid in the diet without negative health effects (Kinsella et al. 1985; Rumsey et al. 1991; Andersen et al. 2006). In contrast, De la Higuera et al. (1981) and Sanchez-Muniz et al. (1982) found that feeding yeast, *Wickerhamomyces anomalus*, at 81 % of the diet resulted in harmful levels of kidney uric acid and haemolytic anaemia in rainbow trout. Despite the ability of fish to degrade uric acid, catabolism of uric acid and its precursors generates hydrogen peroxide, a reactive oxygen species that can damage erythrocytes and cell membranes if levels of antioxidants are insufficient (Jain 1993; Berg et al. 2015). Hydrogen peroxide forms harmful hydroxyl radicals in the presence of iron or copper unless hydrogen peroxide is reduced to water by catalase or glutathione peroxidase, using glutathione as a substrate (Buetler et al. 2004). Therefore, an upper limit to dietary yeast inclusion may exist based on nucleic acid content and the anti-oxidation capacity of fish.

Aside from negative effects, yeast cell walls can be disrupted due to heat and pressure during feed extrusion (Nasseri et al. 2011) and release compounds, such as β-glucans and mannan-oligosaccharides, that have been shown in previous studies to stimulate immune function and reduce stress response in fish, as reviewed by Ringø et al. (2011) and Meena et al. (2013). Those reviews concluded that feeding fish β-glucans and other immunostimulants typically results in increased respiratory burst and macrophage, lysozyme and leucocyte activities that boost resistance to infections and stress. The reviews also noted the lack of research on short-term and haematological effects of these compounds, especially when derived from intact yeast.

Dorsal aorta (DA) cannulation was first described by Conte et al. (1963) for the purpose of evaluating short-term variations in blood and plasma parameters in fish. Since then, multiple modifications to DA cannulation surgery and blood collection procedures have been made to reduce stress and evaluate voluntarily feeding fish (Soivio et al. 1975; Zohar 1980; Gamperl et al. 1994; Kiessling et al. 1995; Lo et al. 2003; Djordjevic et al. 2012). For example, combinations of anaesthetics, cannula abolishing sutures, reduced surgery time, tank design improvements and avoidance of secondary infections have improved DA cannulation (Zahl et al. 2012). Based on these advances, many studies have been able to sample blood from voluntarily feeding Atlantic salmon (*Salmo salar*) and evaluate short-term effects of feed ingredients and supplements (Hamre et al. 2001; Kiessling et al. 2003; Sunde et al. 2003; Olsen et al. 2005; Kiessling et al. 2006, 2009; Djordjevic et al. 2012). In contrast, most studies with DA-cannulated rainbow trout have used force-feeding (Ok et al. 2001; Karlsson et al. 2006; Eliason et al. 2010), which can stress and suppress metabolic pathways (Vijayan et al. 1991; Cooper and Wilson 2008). Hence, achieving voluntary feeding in DA-cannulated rainbow trout is essential to evaluate short-term effects of diet and stress on blood physiology.

The main objective of this study was to determine the effects of feeding high levels of yeast in the diet on whole blood parameters of DA-cannulated rainbow trout. A secondary objective was to distinguish between diet and stress effects of feeding yeasts and inducing an acute stressor on DA-cannulated rainbow trout.

## Materials and methods

### Fish and facilities

**Fig. 1:**
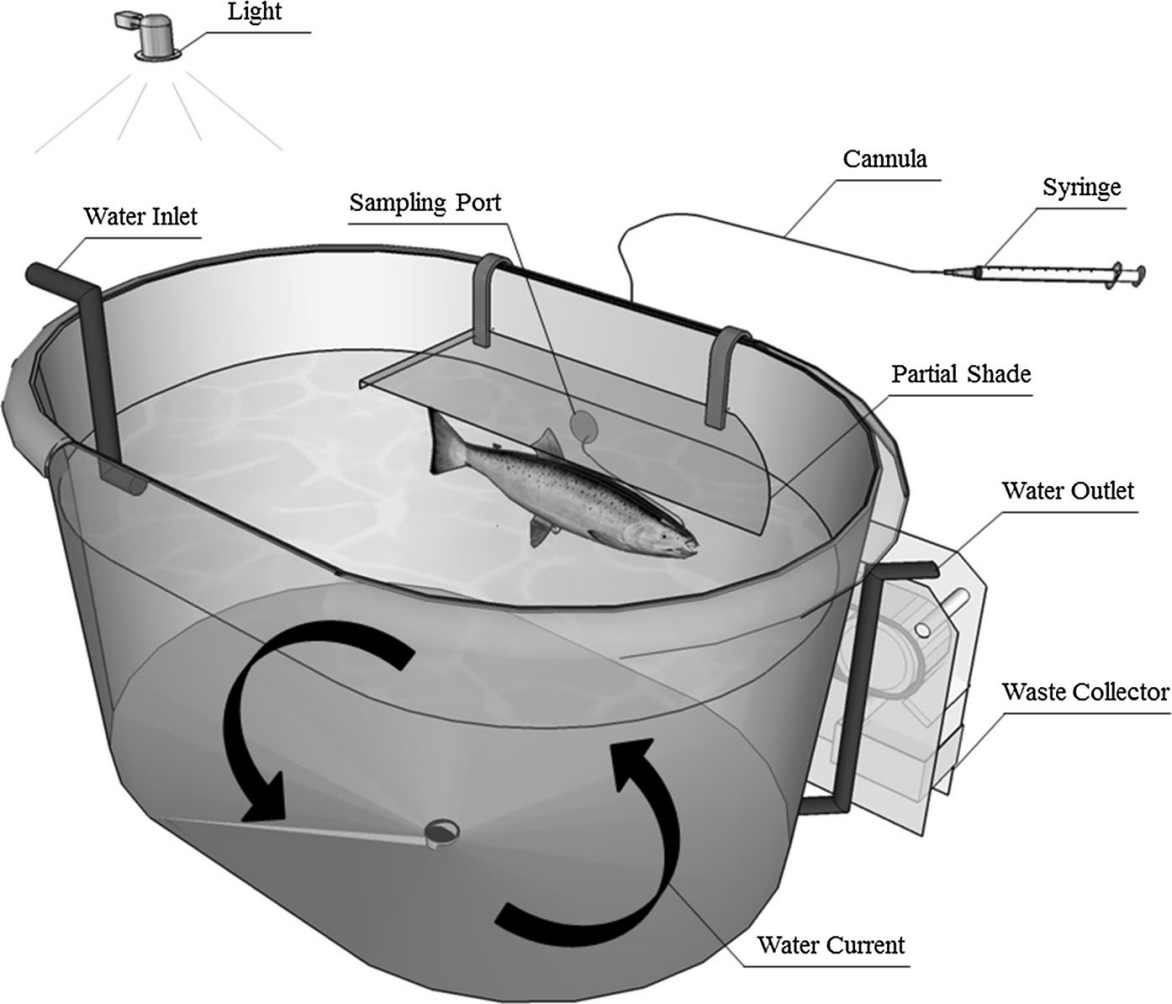
Illustration of the tank design where the position of the light, shade and water outlet directed the dorsal aorta-cannulated rainbow trout adjacent to a sampling port for undisturbed blood collection

The experiment was carried out in the Aquatic Facility of the Veterinary Medicine and Animal Science Centre at the Swedish University of Agricultural Sciences (Uppsala, Sweden). Rainbow trout were acquired from a commercial producer, Vilstena fiskodling AB (Fjärdhundra, Sweden), and raised in groups in 200-L oval tanks. The groups were reduced periodically to one fish per tank that weighed 849 ± 199 g (mean ± SD). Each tank was equipped with a partial shade (80 × 20 cm), LED light and water flow at a rate of approximately 5 L min^−1^ (illustrated in Fig. 1). The combination of shade, light and water outlet positioned the fish voluntarily beside a collection port (5 cm diameter), which permitted undisturbed sampling. The tank system was flow-through and sourced with municipal freshwater that was analysed for dissolved oxygen (10.4 ± 0.7 mg L^−1^), temperature (14.7 ± 0.2 °C) and pH (8.09 ± 0.05) on a weekly basis. Fish were acclimatised to a 14:10 light cycle (lights on at 08:00) in order to collect 0- and 12-h samples and were fed a commercial diet for at least 5-day post-surgery prior to the beginning of the trial. The present study was performed in compliance with laws and regulations on the use of animals for research purposes in Sweden, which is overseen by the Swedish Board of Agriculture (permit reference C74-14).

### Dorsal aorta cannulation procedure

The DA cannulation was performed according to Soivio et al. (1975), with modifications devised by Kiessling et al. (1995), (2003) and Djordjevic et al. (2012). In brief, each fish was sedated with 1 mg L^−1^ metomidate (Aquacalm, Western Chemical Inc., Ferndale, USA) according to Kreiberg and Powell (1991), placed in an aerated bath and anesthetised with 80 mg L^−1^ tricaine methane sulphonate (MS222; Finquel, Scan Aqua AS, Årnes, Norway) buffered with sodium bicarbonate to prevent pH changes. The anesthetised fish was transferred to a surgery bath that recirculated 60 mg L^−1^ buffered MS222 over the gills of the fish to maintain anaesthesia. Local anaesthetics of 0.1 mL lidocaine with adrenaline (20 mg mL^−1^; Xylocaine^®^, AstraZeneca, Södertälje, Sweden) were injected into the proximal roof of the mouth at the snout incision site, and 0.1 mL lidocaine without adrenaline (5 μg mL^−1^; Haukeland Sykehusapotek, Bergen, Norway) was injected into the distal roof near the dorsal aorta incision site. A hole was punctured through the snout with a sterile needle in order to insert a 4-cm piece of 180 polyethylene (PE) tubing to act as a stopper. A 100-cm piece of 90 PE tubing was heated to make a bulb 5 cm from the end to prevent the cannula from dislodging. The tip was narrowed and two microscopic holes were cut out to prevent the cannula from clogging. The cannula was heparinised with 150 IU Na-heparin (LEO Pharma AS, Ballerup, Denmark) in saline (0.9 % NaCl) and then inserted into the DA of the fish via a guide wire after making an incision between the first and second set of gill arches. The cannula was drawn through the larger tubing previously inserted in the snout, injected with heparin–saline solution and the end was heat-sealed. An 80-cm piece of 180 PE tubing was added to the cannula exterior to prevent damage from fish bites. Each fish was weighed after the surgical procedure and returned to the tank after gently spreading mucus from untouched skin to any affected areas of the fish to reduce the risk of infection. Each fish was gently guided around the tank to increase water flow over the gills and reduce recovery time. All fish resumed feeding within hours after surgery.

### Diets and feeding

In total, 15 DA-cannulated fish were fed a control diet of 30 % fishmeal (FM) and two test diets of yeasts: *S. cerevisiae* (SC) or a 70:30 mix of *W. anomalus* and *S. cerevisiae* (WA). In the SC and WA diets, 60 % of fish meal protein was replaced with yeast protein to achieve 380 g kg^−1^ digestible protein (dry matter; DM) based on 95 and 86 % digestibility coefficients for fish meal and yeast (NRC 2011; Langeland et al. 2016; Vidaković 2015). The diets were produced by extrusion at the Natural Resources Institute Finland (Laukaa, Finland) with a twin-screw extruder (3 mm die, BC-45 model, Clextral, Creusot Loir, France). Extruded pellets were air-dried overnight at 60 °C and then sprayed with lipids using a vacuum coater (Pegasus PG-10VC, Dinnissen, Sevenum, Netherlands). For diet and proximate composition, see Table 1.

**Table 1:** Composition of the fish meal control diet (FM), a diet with 60 % of fish meal replaced with *Saccharomyces cerevisiae* yeast (SC) and a diet with 60 % of fish meal replaced with *Wickerhamomyces anomalus* and *S. cerevisiae* yeast (WA)

	Diet		
	FM	SC	WA
Ingredients (g kg^−1^ as-is basis)			
Fish meal^a^	300	120	120
*S. cerevisiae* yeast^b^	–	321	–
*W. anomalus*/*S. cerevisiae* yeast^c^	–	–	355
Soy protein concentrate	135	135	135
Wheat gluten	120	120	120
Wheat starch	100	10	0
Wheat meal	60	60	60
Fish oil	110	125	124
Rapeseed oil	50	50	50
Titanium oxide	5	5	5
Mineral-vitamin premix	15	15	15
Monocalcium phosphate	10	10	10
Cellulose	93	24	0
l-methionine	2	5	6
Proximate composition (g kg^−1^ DM basis)			
Dry matter (%)	92	91	93
Crude protein^d^	425	454	463
Crude lipid	196	203	186
Gross energy (MJ kg^−1^)	24	24	24
Neutral detergent fibre	114	45	25
Ash	68	63	62

Modified from Vidaković (2015)

^a^Low-temperature dried Icelandic capelin meal (Rasioagro Ltd, Rasio, Finland); 90 % dry matter; 710 g kg^−1^ crude protein; 117 g kg^−1^ crude lipid; 80 g kg^−1^ ash

^b^Dried yeast meal (Jästerbolaget AB, Uppsala, Sweden); 94 % dry matter; 435 g kg^−1^ crude protein; 9 g kg^−1^ crude lipid; 58 g kg^−1^ ash; 78 g kg^−1^ nucleic acids

^c^Dried yeast meal with 70:30 *W. anomalus* to *S. cerevisiae* (Jästerbolaget AB, Uppsala, Sweden); 93 % dry matter; 391 g kg^−1^ crude protein; 8 g kg^−1^ crude lipid; 65 g kg^−1^ ash; 76 g kg^−1^ nucleic acids

^d^Diets balanced on a digestible protein content of 390 g kg^−1^

Each diet was analysed for crude protein (% N × 6.25) using a Kjeltec 2020 digester and 2400 analyser (FOSS Analytical A/S, Hilleröd, Denmark) according to the Kjeldahl method (Nordic Committee on Food Analysis 1976), crude lipids using a Soxtec HT 1043 extractor (FOSS Analytical A/S, Hilleröd, Denmark) without acid hydrolysis according to the manufacturer (ANKOM Technology, Macedon, NY, USA), gross energy using a Parr 6300 isoperibol calorimeter (Parr Instrument Company, Moline, IL, USA) and nutrient detergent fibre according to the Amylase Neutral Detergent method (Mertens 2002). Diets and test ingredients were analysed for ash using an incinerator at 550 °C for 3 h and buffering capacity by titrating 1 g with 0.1 M lactic acid until the pH stabilised at 4.0 for 10 min (McDonald Henderson 1962). Yeast ingredients were analysed for total nucleic acids (TNA) using a sonicator at 20 Wm^−2^ for 3 min in 0.5 N perchloric acid and measuring absorbance at 260 nm (TNA = OD_260nm_ × 45.5) (Zachleder 1984).

Five fish per diet were fed at 1 % body weight (BW) via automatic belt feeders (Hølland teknologi, Sandnes, Norway) once per day (i.e. 10:00 to 11:00) in a 3 × 3 randomised, cross-over design for 3 weeks. Feed waste from each tank was collected continuously using belt collectors (Hølland teknologi, Sandnes, Norway), weighed daily and pooled weekly. Feed and feed waste were analysed for DM content after drying at 103 °C for 16 h and then used to calculate feed intake: (Feed Fed DM − (Feed Waste DM/Recovery))/Feed DM, where recovery is the percentage of feed recovered after collection, according to Helland et al. (1996). Blood samples from fish with feed intake less than 0.2 % BW day^−1^ were considered non-representative and rejected.

### Blood sampling and stress procedures

Blood samples were taken from the cannula of each fish at 0 (30–60 min before feeding), 3, 6, 12 and 24 h after feeding on the 7th day of each week. In brief, the free-floating cannula was gently drawn through the collection port on the side of the tank using a thin hook. The cannula was cut, and the saline and first 0.1 mL of blood was discarded. Using a new heparinised syringe, 0.35 mL of blood was withdrawn from the cannula and then replaced with saline containing 150 IU Na-heparin before heat sealing and returning the cannula to the tank. After whole blood analyses (below), samples were immediately centrifuged at 500*g* for 3 min and the plasma was transferred to cryotubes, stored at −20 °C and later transferred to a −80 °C freezer.

To test for diet–stress interactions, blood samples were collected from fish fed for a fourth week on the same diet as in week three and then exposed to an acute stressor. Fish were removed from the water, held in a net beside the tank for 1 min and then returned. Fish swimming behaviour was monitored and showed that feed regurgitation was negligible, as the stress was induced 30 min after feeding to minimise vomiting (i.e. at 11:30). The first sample collected after netting occurred at 3-h post-prandium or 30-min post-stress (i.e. 12:00).

### Blood and plasma analyses

To determine effects on blood electrolytes and gases, blood was injected into EC8+ cassettes inserted into an i-STAT portable analyser (i-STAT Corporation, East Windsor, NJ, USA) that measured sodium (Na), potassium (K), glucose, pH, partial carbon dioxide (PCO_2_), total carbon dioxide (TCO_2_), bicarbonate (HCO_3_), base excess (BE) and haemoglobin (Hb). Previous studies have validated i-STAT analyses against conventional methodologies for different fish species (Harrenstien et al. 2005; Harter et al. 2014).

To determine effects on erythrocytes, microcapillary tubes of blood were centrifuged at 12,000*g* for 5 min and haematocrit (Hct) and leucocrit (Lct) levels were measured using linear and microscopic rulers. Red blood cells (RBC) were counted at 400× magnification in five 1 mm^2^ squares within a Bürker haemocytometer (Glaswarenfabrik Karl Hecht GmbH & Co KG, Sondheim, Germany) after 1:20 dilution with Turk’s solution (Stoskopf 1993). Erythrocyte area was measured from blood smears that were fixed and stained with methanol and Giemsa (Vázquez and Guerrero 2007). Four images at 400× magnification were taken from the periphery of the smear, and 10 random erythrocytes were automatically measured using NIS Elements Basic Research software (Nikon Instruments Europe BV, Amsterdam, Netherlands). Lastly, erythrocyte indices were calculated based on mean corpuscular volume (MCV = Hct/RBC × 10), mean corpuscular haemoglobin (MCH = Hb/RBC × 10) and mean corpuscular haemoglobin concentration (MCHC = Hb/Hct × 100) (Stoskopf 1993).

As a stress indicator, cortisol was analysed from plasma using 96-well, multi-species ELISA kits (DetectX©, Arbor Assays, Ann Arbor, MI, USA) at 1:25 and 1:100 dilutions with assay buffer for unstressed and stressed fish. Ten plasma samples from unstressed and stressed samples were cross-referenced between the ELISA and RIA methods for cortisol and produced a covariance of 8.6 ± 2.6 % (mean ± SE).

### Statistical analyses

Data were analysed using Linear Mixed Effects models (Bates et al. 2015) with R^®^ version 3.2.2 software (R-Core-Team 2015). Fixed effects included in the models were diet, sampling time, feed intake (% BW day^−1^) and fish weight, determined to be significant using ANOVA. In addition, the following terms were included in the models: random effects of fish and week to account for individual variation, interaction between diet and hour to account for hourly variation in blood parameters, and a correlation between hour and fish-week to account for repeated sampling. To determine diet–stress effects, unstressed fish from week 3 were compared with stressed fish from week 4 fed the same diet. Significant diet and stress effects were determined using post hoc Least Square Means tests (Lenth 2014) with Tukey adjustment for pair-wise comparisons. Differences between buffering capacity and ash content were determined using paired Student’s *t* test. Standardised residuals of all models were tested for normality by normal probability plots, *p* < 0.05 was considered statistically significant, and *p* < 0.15 was considered a tendency.

## Results

In 4 weeks, fish achieved a mean weight gain of 161 ± 107 g or 19 ± 12 % (mean ± SD) and fish fed diets FM, SC and WA achieved similar mean feed intakes of 0.84 ± 0.22, 0.89 ± 0.23 and 0.81 ± 0.24 % BW day^−1^ (*p* = 0.589). For fish with feed intakes less than 0.2 % BW day^−1^, 14 out of 60 blood samples were excluded from analyses. There were no mortalities; however, five fish were replaced due to non-functioning cannulas.

### Electrolyte, blood gases and pH analyses

**Fig. 2:**
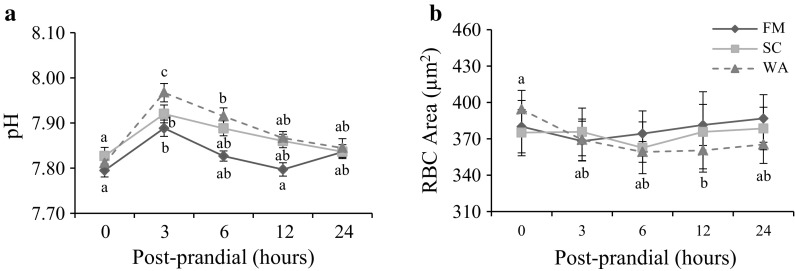
Post-prandial whole blood values (mean ± SE) of **a** pH and **b** red blood cell (RBC) size in rainbow trout fed diets containing fish meal (FM, *filled diamond*), *Saccharomyces cerevisiae* yeast (SC, *filled square*) and *Wickerhamomyces anomalus* and *S. cerevisiae* yeast (WA, *filled triangle*). Values within diet followed by different *lower case letters* differ significantly (*p* < 0.05)

**Fig. 3:**
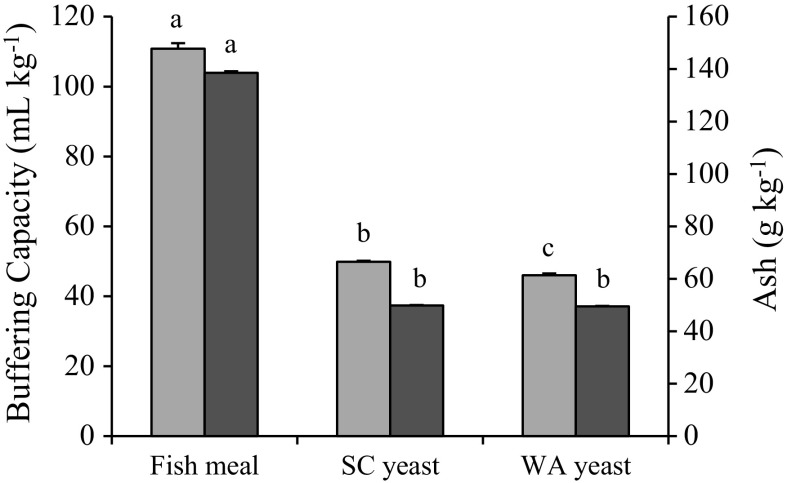
Buffering capacity (*light grey*) and ash content (*dark grey*) on a kg^−1^ DM basis of the fish meal, *Saccharomyces cerevisiae* yeast (SC) and *Wickerhamomyces anomalus* and *S. cerevisiae* yeast (WA) ingredients. Values for each parameter followed by different *lower case letters* differ significantly (*p* < 0.05)

Levels of pH, TCO_2_, HCO_3_ and BE increased significantly in fish fed both yeast-based diets compared with fish fed the FM diet (Table 2; Fig. 2). The highest measured levels of pH, TCO_2_, HCO_3_ and BE occurred at the first post-prandial sample (3 h), whereas Na and PCO_2_ levels did not change significantly over time. Glucose and K showed different post-prandial profiles between diets, as the highest values were at 6 and 0 h (continually decreased) for fish fed the FM diet, while the highest values were later (i.e. 12 and 3 h) for the SC and WA diets. Fish fed yeast-based diets showed a tendency for an increase in K compared with fish fed the FM diet. Acid titration and ashing of the test ingredients showed that buffering capacity and ash content of fish meal were at least twofold higher than those of both yeasts (Fig. 3).

**Table 2:** Electrolyte and gas values (mean ± SE) in blood sampled from rainbow trout before and after they were fed diets containing fish meal (FM; *n* = 12), *Saccharomyces cerevisiae* yeast (SC; *n* = 9) or *Wickerhamomyces anomalus* and *S. cerevisiae* yeast (WA; *n* = 13)

Variable	Diet	Time after feeding (h)^a^					*p* value^b^
		0	3	6	12	24	
Electrolytes							
Na (mmol L^−1^)	FM	151.7 ± 0.5	151.5 ± 0.6	151.5 ± 0.5	151.6 ± 0.6	151.2 ± 0.6	
	SC	150.3 ± 0.5	150.2 ± 0.6	150.2 ± 0.4	150.1 ± 0.6	150.6 ± 0.4	0.179
	WA	151.0 ± 0.4	150.5 ± 0.5	150.5 ± 0.7	150.6 ± 0.6	150.9 ± 0.8	0.169
K (mmol L^−1^)	FM	2.81 ± 0.10a	2.74 ± 0.12ab	2.59 ± 0.08ab	2.52 ± 0.08b	2.56 ± 0.09ab	
	SC	2.80 ± 0.07ab	3.04 ± 0.12b	2.97 ± 0.09ab	2.73 ± 0.09a	2.88 ± 0.10ab	0.076
	WA	2.83 ± 0.10ab	3.01 ± 0.09b	2.79 ± 0.10ab	2.63 ± 0.10a	2.78 ± 0.11ab	0.136
Glucose (mmol L^−1^)	FM	3.43 ± 0.12ab	4.08 ± 0.21c	4.33 ± 0.26c	4.01 ± 0.26b	3.40 ± 0.21a	
	SC	3.24 ± 0.09a	3.91 ± 0.15a	4.08 ± 0.18b	4.31 ± 0.32b	3.31 ± 0.16a	0.449
	WA	3.23 ± 0.11a	3.77 ± 0.01ab	4.30 ± 0.15bc	4.66 ± 0.29c	3.15 ± 0.12a	0.934
pH	FM	7.79 ± 0.02a	7.89 ± 0.02b	7.83 ± 0.01ab	7.80 ± 0.02a	7.84 ± 0.01ab	
	SC	7.83 ± 0.02a	7.92 ± 0.02b	7.89 ± 0.02ab	7.86 ± 0.02ab	7.84 ± 0.02ab	0.048
	WA	7.81 ± 0.02a	7.97 ± 0.02c	7.91 ± 0.02b	7.87 ± 0.02ab	7.84 ± 0.02ab	0.001
Blood gases							
pCO_2_ (mmHg)	FM	5.9 ± 0.1	5.6 ± 0.1	5.8 ± 0.1	5.7 ± 0.1	5.6 ± 0.1	
	SC	5.7 ± 0.1	5.9 ± 0.2	5.8 ± 0.1	5.9 ± 0.1	5.8 ± 0.1	0.843
	WA	5.7 ± 0.1	5.6 ± 0.1	5.9 ± 0.1	5.7 ± 0.2	5.6 ± 0.2	0.971
TCO_2_ (mmol L^−1^)	FM	9.2 ± 0.3	11.0 ± 0.5	9.8 ± 0.4	9.1 ± 0.4	9.6 ± 0.3	
	SC	9.7 ± 0.5a	12.4 ± 0.9b	11.2 ± 0.4ab	10.8 ± 0.5ab	10.1 ± 0.4ab	0.028
	WA	9.3 ± 0.4a	13.1 ± 0.7c	12.2 ± 0.6bc	10.5 ± 0.4ab	9.8 ± 0.3a	0.001
HCO_3_ (mmol L^−1^)	FM	9.0 ± 0.3	10.9 ± 0.5	9.7 ± 0.4	8.9 ± 0.4	9.4 ± 0.3	
	SC	9.5 ± 0.5a	12.2 ± 0.8b	11.0 ± 0.4ab	10.6 ± 0.5ab	9.9 ± 0.4ab	0.028
	WA	9.1 ± 0.4a	12.9 ± 0.7c	12.1 ± 0.6b	10.3 ± 0.4ab	9.7 ± 0.3a	0.001
BE (mmol L^−1^)	FM	−11.8 ± 0.6a	−8.9 ± 0.8b	−11.0 ± 0.5ab	−12.1 ± 0.5a	−11.1 ± 0.4ab	
	SC	−10.9 ± 0.7a	−7.0 ± 1.1b	−8.7 ± 0.6ab	−9.5 ± 0.6ab	−10.6 ± 0.6a	0.020
	WA	−11.5 ± 0.5a	−5.8 ± 1.0c	−7.3 ± 0.9bc	−9.8 ± 0.6ab	−10.8 ± 0.6a	0.001

*Na* sodium, *K* potassium, *pCO*_*2*_ partial carbon dioxide, *TCO*_*2*_ total carbon dioxide, *HCO*_*3*_ bicarbonate, *BE* base excess

^a^Values within variable and diet followed by different lower case letters differ significantly (*p* < 0.05)

^b^ *P* values for each mean variable of either the SC or WA diet compared with the FM diet based on linear mixed effects models that included diet, time, feed intake and weight as fixed effects and fish and week as random effects

### Haemotological analyses

Levels of Hct, Lct, RBC and Hb were not significantly different between diets and reached their lowest values at 12 or 24 h post-prandium (Table 3). No differences were observed for erythrocyte indices, except for MCH levels between fish fed the FM and WA diets, while there was a tendency for an increase in fish fed the SC and FM diets (Table 3; Fig. 2). Erythrocyte size was not significantly different between all diets, although area decreased significantly between 0 and 12 h samples in fish fed the WA diet (Table 3).

**Table 3:** Haematology, red blood cell indices and hormone values (mean ± SE) in blood sampled from rainbow trout before and after they were fed diets containing fish meal (FM; *n* = 12), *Saccharomyces cerevisiae* yeast (SC; *n* = 9) or *Wickerhamomyces anomalus* and *S. cerevisiae* yeast (WA; *n* = 13)

Variable	Diet	Time after feeding (h)^a^					*p* value^b^
		0	3	6	12	24	
Haematology							
Hct (%)	FM	19.8 ± 0.9ab	21.1 ± 1.2a	18.3 ± 0.7b	17.6 ± 0.8b	17.5 ± 0.8b	
	SC	20.2 ± 0.8	21.2 ± 1.2	19.3 ± 0.9	18.5 ± 1.5	18.6 ± 1.2	0.920
	WA	21.7 ± 1.4a	21.0 ± 0.9ab	19.2 ± 1.1ab	19.3 ± 1.2ab	18.4 ± 0.7b	0.681
Lct (%)	FM	1.1 ± 0.05a	0.9 ± 0.06ab	0.9 ± 0.06ab	0.8 ± 0.07b	1.0 ± 0.08a	
	SC	0.9 ± 0.1	1.0 ± 0.1	0.9 ± 0.1	0.7 ± 0.1	0.8 ± 0.1	0.855
	WA	1.0 ± 0.1	0.9 ± 0.1	0.9 ± 0.1	0.8 ± 0.1	0.9 ± 0.1	0.982
Hb (g dL^−1^)	FM	5.8 ± 0.2	5.6 ± 0.3	5.4 ± 0.2	5.0 ± 0.2	5.1 ± 0.2	
	SC	6.1 ± 0.4	6.0 ± 0.3	5.8 ± 0.3	5.7 ± 0.5	5.3 ± 0.5	0.602
	WA	6.2 ± 0.4a	6.3 ± 0.2a	5.8 ± 0.3ab	5.8 ± 0.4ab	5.1 ± 0.2b	0.351
RBC (10^6^ cells µL^−1^)	FM	0.78 ± 0.06	0.77 ± 0.04	0.72 ± 0.04	0.70 ± 0.04	0.68 ± 0.03	
	SC	0.74 ± 0.05	0.77 ± 0.06	0.74 ± 0.05	0.74 ± 0.06	0.65 ± 0.04	0.838
	WA	0.70 ± 0.05	0.73 ± 0.04	0.75 ± 0.04	0.72 ± 0.05	0.68 ± 0.04	0.614
RBC area (µm^2^)	FM	380.0 ± 21.6	368.1 ± 16.4	374.3 ± 18.7	381.4 ± 16.9	386.8 ± 19.6	
	SC	375.1 ± 19.0	375.7 ± 19.7	362.6 ± 21.4	375.7 ± 33.1	378.5 ± 17.5	0.921
	WA	394.3 ± 15.7a	369.1 ± 17.1ab	359.2 ± 8.5ab	360.4 ± 15.2b	365.3 ± 15.6ab	0.999
Erythrocyte Indices							
MCV (fL cell^−1^)	FM	267.2 ± 19.1	296.9 ± 42.1	261.9 ± 15.9	260.8 ± 17.9	260.2 ± 13.9	
	SC	282.5 ± 16.9	291.9 ± 30.8	273.8 ± 24.3	253.6 ± 14.0	294.0 ± 20.5	0.704
	WA	325.5 ± 22.6	289.9 ± 8.7	259.0 ± 9.8	272.1 ± 9.3	278.1 ± 11.5	0.476
MCH (pg cell^−1^)	FM	78.6 ± 5.4	75.6 ± 6.1	77.5 ± 4.9	74.3 ± 6.4	75.4 ± 3.8	
	SC	85.3 ± 5.7	82.6 ± 9.7	82.6 ± 7.9	77.6 ± 5.3	84.2 ± 9.0	0.108
	WA	93.9 ± 7.6	87.5 ± 3.2	78.7 ± 3.0	81.6 ± 3.7	78.5 ± 4.5	0.029
MCHC (g dL^−1^)	FM	29.7 ± 1.0	26.7 ± 1.0	29.6 ± 0.5	28.3 ± 0.7	29.3 ± 1.3	
	SC	30.2 ± 0.8	28.1 ± 0.4	30.1 ± 0.6	30.6 ± 1.3	28.1 ± 1.2	0.489
	WA	28.7 ± 0.7	30.2 ± 0.8	30.5 ± 0.7	29.9 ± 0.5	28.2 ± 0.9	0.214
Hormones							
Cortisol (ng mL^−1^)	FM	3.1 ± 0.9	3.5 ± 0.9	3.4 ± 1.1	6.4 ± 2.2	3.5 ± 0.9	
	SC	2.6 ± 0.6a	1.8 ± 0.6a	2.4 ± 0.6a	7.1 ± 1.4b	2.9 ± 0.7a	0.895
	WA	3.3 ± 0.6	3.6 ± 1.0	4.0 ± 1.4	6.6 ± 1.7	3.1 ± 0.6	0.967

*Hct* haematocrit, *Lct* leucocrit, *Hb* haemoglobin, *RBC* red blood cells, *MCV* mean corpuscular volume, *MCH* mean corpuscular haemoglobin, *MCHC* mean corpuscular haemoglobin concentration

^a^Values within variable and diet followed by different lower case letters differ significantly (*p* < 0.05)

^b^ *P* values for each mean variable of either the SC or WA diet compared with the FM diet based on linear mixed effects models that included diet, time, feed intake and weight as fixed effects and fish and week as random effects

### Acute stress response

Whole blood parameters and plasma cortisol concentration showed no significant differences between stressed fish fed the FM and yeast diets, and therefore values from fish fed all three diets were pooled to compare stressed and non-stressed fish. In contrast to unstressed fish, levels of pH, TCO_2_, HCO_3_ and BE decreased significantly in stressed fish, with the lowest levels occurring at 0.5 h post-stress (Table 4). In addition, Hct and Lct were highest at 3.5 h post-stress, whereas consistent decreases over time were observed for unstressed fish. Lastly, significant increases in cortisol and glucose occurred at 0.5 and 3.5 h post-stress, with cortisol concentration increasing more than 20-fold (Fig. 5).

**Table 4:** Whole blood and plasma cortisol values (mean ± SE) in rainbow trout sampled before and after exposure to a 1-min netting stressor (Pooled diets; *n* = 12)

Variable	Time after stress (h)^a^					*p* value^b^
	0	0.5	3.5	9.5	21.5	
Glucose (mmol L^−1^)	3.2 ± 0.1a	5.0 ± 0.2bc	5.6 ± 0.5c	5.9 ± 0.5c	4.4 ± 0.4ab	0.012
pH	7.79 ± 0.01a	7.62 ± 0.03b	8.00 ± 0.03c	7.93 ± 0.01 cd	7.84 ± 0.02ad	0.032
Hct (%)	19.7 ± 0.8ab	23.4 ± 0.9c	20.0 ± 0.7bc	18.1 ± 1.3b	16.7 ± 0.7a	0.399
Lct (%)	1.05 ± 0.09a	1.15 ± 0.10ab	0.83 ± 0.05c	0.77 ± 0.04c	0.99 ± 0.08ac	0.674
TCO_2_ (mmol L^−1^)	8.6 ± 0.3a	5.9 ± 0.3b	13.7 ± 1.0c	11.7 ± 0.4 cd	9.9 ± 0.4ad	0.038
HCO_3_ (mmol L^−1^)	8.5 ± 0.3a	5.7 ± 0.3b	13.6 ± 1.0c	11.6 ± 0.3 cd	9.8 ± 0.4ad	0.038
BE (mmol L^−1^)	−12.3 ± 0.4a	−17.3 ± 0.6b	−5.0 ± 1.3c	−8.0 ± 0.5 cd	−10.7 ± 0.6ad	0.028
Cortisol (ng mL^−1^)	6.4 ± 1.5a	129.2 ± 27.3b	38.5 ± 18.2a	15.0 ± 2.7a	6.2 ± 1.3a	0.014

*Hct* haematocrit, *Lct* leucocrit, *TCO*_*2*_ total carbon dioxide, *HCO*_*3*_ bicarbonate, *BE* base excess

^a^Values within variable followed by different lower case letters differ significantly (*p* < 0.05)

^b^ *P* values for each mean variable of either the SC or WA diet compared with the FM diet based on linear mixed effects models that included diet, time, feed intake and weight as fixed effects and fish and week as random effects

## Discussion

### Buffering capacity

Significantly increased levels of blood pH, TCO_2_, HCO_3_ and BE in fish fed the SC and WA diets (Table 2) indicated that the yeast diets induced higher alkaline tides than the FM diet. Alkaline tide is caused by efflux of HCO_3_ into the blood to prevent alkalinisation of the parietal cells after secretion of HCl into the stomach lumen during digestion (Niv and Fraser 2002). Bucking and Wood (2008) and Cooper and Wilson (2008) found similar increases in pH, HCO_3_ and BE in DA-cannulated rainbow trout fed diets based on fish meal and suggested that high buffering capacity of fish meal influenced alkaline tide. Fish meal has been shown to have one of the highest buffering capacities among feedstuffs, and this characteristic has been correlated to high cation and ash content (Jasaitis et al. 1987; Lević et al. 2005; Montañez-Valdez et al. 2013). The present study confirmed that fish meal had more than twofold higher levels of ash and buffering capacity compared with both yeast ingredients (Fig. 3). Lower buffering capacity has been shown to have beneficial effects on protein metabolism and intestinal microbiota due to increased stomach pH (Eckel et al. 1992; Gabert and Sauer 1994). A study by our research group on yeast-induced changes to intestinal pH and microbiota is ongoing.

### Erythrocyte size

**Fig. 4:**
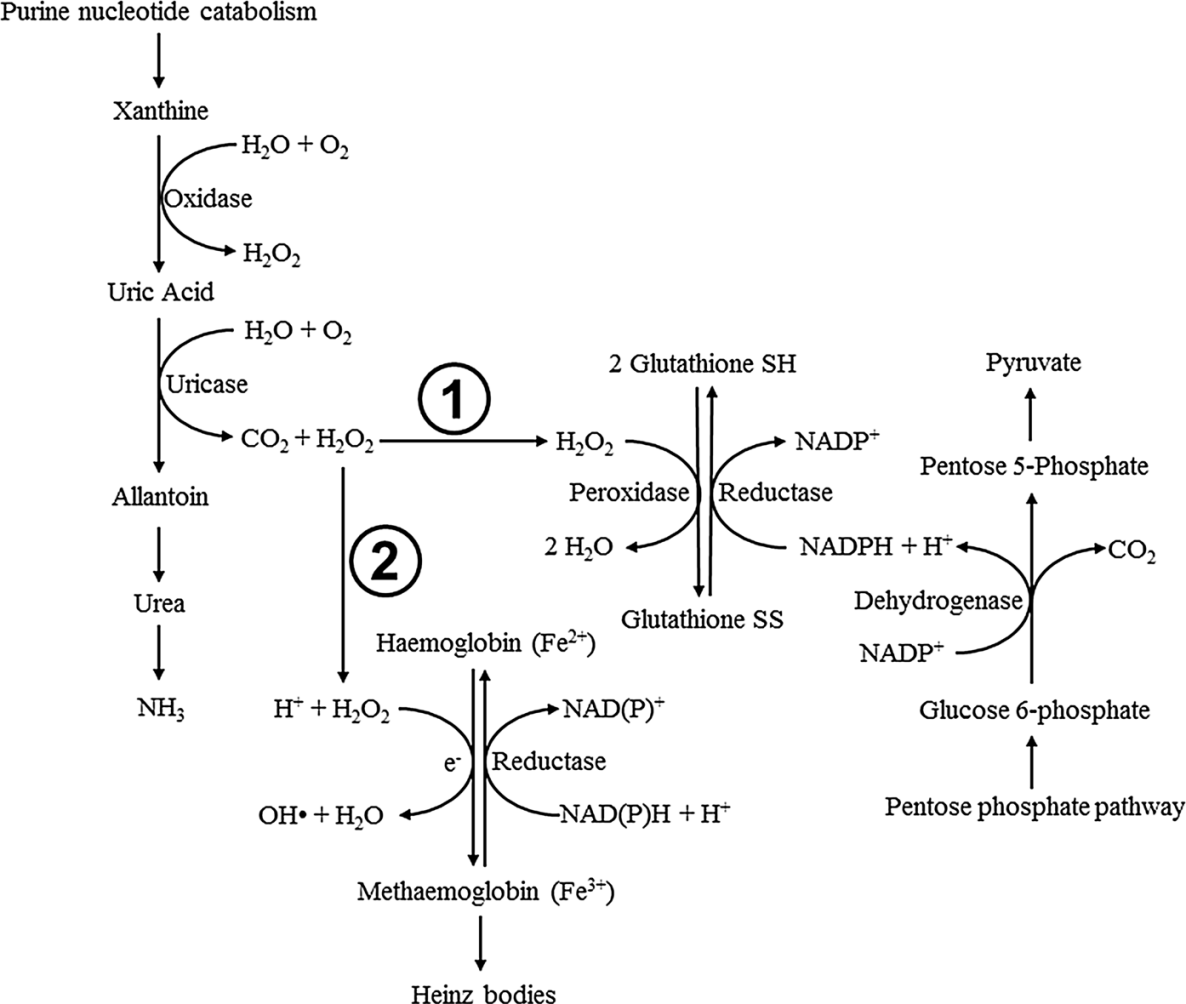
Simplified diagram of purine nucleotide catabolism in fish, resulting in production of hydrogen peroxide (H_2_O_2_) that can be: *(1)* reduced to H_2_O by glutathione peroxidase given adequate glutathione SH:SS ratio maintained by the pentose phosphate pathway, or *(2)* used to oxidise haemoglobin to methaemoglobin molecules that link to form Heinz bodies, modified from Sanchez-Muniz et al. (1982) and Berg et al. (2015)

Fish fed the WA diet had significantly smaller erythrocytes between 0 and 12 h after feeding (Table 3), which is indicative of haemolytic anaemia (Jain 1993). In fish, damage to erythrocytes results in increased production and replacement with smaller immature erythrocytes to compensate for losses in oxygen transport (Stoskopf 1993; Clauss et al. 2008; Hrubec and Smith 2010). Sanchez-Muniz et al. (1982) found that rainbow trout fed diets containing 81 % *W. anomalus* had reduced erythrocyte size and lowered peroxidase activity compared with fish fed a fish meal-based diet. In fish and other monogastrics, catabolism of high levels of purine nucleotides, such as oxidation of xanthine and uric acid, has been shown to produce high levels of hydrogen peroxide that result in erythrocyte damage (Clifford and Story 1976; Goldenberg 1977; Bontemps et al. 1986; Rumsey et al. 1992; Berg et al. 2015). Glutathione and glutathione peroxidase are responsible for diminishing toxic effects of hydrogen peroxide, but if the glutathione concentration and/ or the peroxidase activity is insufficient that can lead to oxidation of haemoglobin to methaemoglobin, Heinz body formation and eventual haemolysis (Mills and Randall 1958; Berg et al. 2015) (see Fig. 4). In addition to deficiencies in glutathione, deficiencies in NADPH or other components of the pentose phosphate pathway required to maintain glutathione levels can lead to decreased haemoglobin protection (Mills and Randall 1958; Berg et al. 2015). Therefore, the haemolysis observed in fish fed yeast diets in the present study suggests that the level of purine nucleotides was too high to be safely metabolised by rainbow trout (Fig. 4).

Haemolytic anaemia can have long-term effects on physiology, such as chronic fatigue, due to insufficient oxygen transport and increased erythropoiesis (Jain 1993). Anaemia effects may explain the reduced fish growth observed in previous studies feeding yeast to rainbow trout, in which above 40 % of fish meal (30 % of the diet) was replaced (Rumsey et al. 1991; Hauptman et al. 2014; Vidaković 2015). Rumsey et al. (1992) showed diets that contained up to 4.1 % yeast-derived nucleic acid (50 % yeast inclusion) did not reduce fish growth, but diets containing 1.5 % adenosine, a purine nucleotide, resulted in decreased growth of rainbow trout. In the present study, yeast ingredients of *S. cerevisiae* and *W. anomalus* mix contributed 2.5 and 2.3 % nucleic acid to SC and WA diets (Table 1), but the level of adenosine was not determined. Interestingly, blood alkalinisation and glucose reductions have been shown to increase purine nucleotide catabolism in vitro (Bontemps et al. 1986; Van den Berghe et al. 1988), whereas both conditions occurred in fish fed yeast diets in the present study and suggests that toxic effects of purine catabolism may have been elevated. However, more research is required to establish the correlation and limit between dietary levels of yeast-derived purine nucleotides with haemolysis and reduced growth for rainbow trout.

### Erythrocyte haemoglobin

In addition to erythrocyte size, there was a significant increase in MCH levels, an indicator of hyperchromic anaemia (Stoskopf 1993), in fish fed the WA diet and a tendency for an increase in fish fed the SC diet (Table 3). In contrast, Sanchez-Muniz et al. (1979) found decreased MCH levels in fish fed a *W. anomalus* diet, indicating hypochromic anaemia. However, oxidation of haemoglobin to methaemoglobin and subsequent formation of Heinz bodies can result in erroneous elevations in MCH and MCHC values (Jain 1993). In comparison with previous studies (Miller et al. 1983; Řehulka et al. 2004), both MCH and MCHC values in the present study were equal to or above the 99th percentile for rainbow trout (Table 3). However, the i-STAT method for haemoglobin determination and the large fish used in the present study may have increased MCH and MCHC values, despite the difference between diets. Microcytic anaemia is typically not associated with hyperchromic conditions since smaller immature erythrocytes should not contain higher levels of haemoglobin (Jain 1993). Higher inclusion of yeast in the WA diet than in the SC diet (Table 1) may have increased the effect of haemolysis on fish, although the non-significant reduction in erythrocyte size and tendency for an increase in MCH in fish fed the SC diet (Table 3) suggest that the SC diet induced haemolysis to a lesser extent. Therefore, the higher MCH levels in the present study may have been artificially increased by formation of Heinz bodies due to haemolysis.

### Acute stress response

**Fig. 5:**
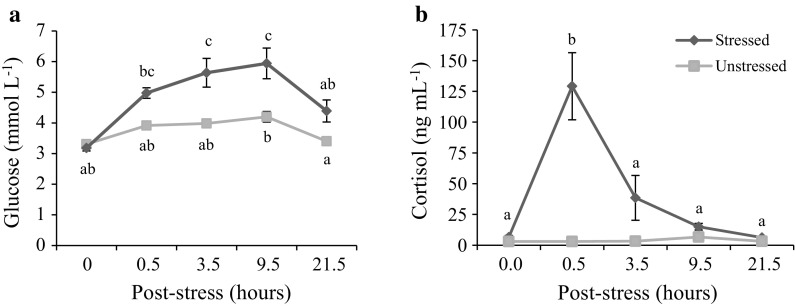
Post-prandial whole blood values (mean ± SE) of **a** glucose, and **b** cortisol in unstressed (*filled square*) and stressed (*filled diamond*) rainbow trout. Values for unstressed or stressed fish followed by different *lower case letters* differ significantly (*p* < 0.05)

Fish showed significantly increased cortisol and glucose levels between 0.5 and 9.5 h post-stress (Fig. 5), but no differences were found between diets. Elevations in cortisol and glucose are used as primary and secondary indicators of stress in fish (Barton and Iwama 1991). In unstressed fish, plasma cortisol levels increased significantly at 12-h post-prandium for all diets (Table 3), which can be explained by diurnal cycling based on circadian rhythm, as shown previously (Holloway et al. 1994; Reddy and Leatherland 2003). Increases in Hct and Lct have also been used as secondary indicators of stress in fish studies (Anderson 1990; Barton and Iwama 1991). In the present study, levels of Hct significantly increased 0.5-h post-stress and then both Hct and Lct levels continually decreased similar to unstressed fish (Table 3 and 4). The slight Hct increase may be a reflection of the low intensity stressor, while continual reductions in Hct and Lct for unstressed fish have been found in previous studies, which attributed the decrease to haemodilution as an effect of repeated blood sampling (Soivio et al. 1972, 1975; Bry and Zohar 1980; Deng et al. 2000). In contrast to Hct, the levels of pH and HCO_3_ significantly decreased immediately after stress (Table 4). This is in agreement with findings in previous studies, which attributed the decrease to elevated gill ventilation and anaerobic processes (Jones and Randall 1979; Turner et al. 1983). Lastly, no effects of the yeast diets were observed on whole blood and cortisol parameters of fish, although more research should be performed on extracted yeast compounds, e.g. β-glucans. Therefore, yeast-based diets did not reduce the acute stress response of fish, but on the other hand these novel protein sources did not induce further stress.

### Voluntary feeding in fish

To the best of our knowledge, this study was the first to examine the effects of diet on whole blood and cortisol parameters in DA-cannulated rainbow trout following voluntary feeding. In contrast with previous studies (Ok et al. 2001; Karlsson et al. 2006; Eliason et al. 2010), force-feeding was avoided in the present study in order to negate stress effects on fish physiology and diet metabolism (Vijayan et al. 1991). For example, Cooper and Wilson (2008) found that blood pH and HCO_3_ were twofold higher in rainbow trout after force-feeding than after voluntary feeding. The success in achieving voluntary feeding in the present study may be attributable to several months of tank acclimatisation with step-wise reductions in fish stocking density, although this was not tested. By eliminating the stress from force-feeding, this study may provide a more realistic scenario of post-prandial whole blood and cortisol parameters in rainbow trout.

Replacing 60 % of fish meal on a digestible protein basis with *S. cerevisiae* and *W. anomalus* mix significantly affected whole blood parameters in rainbow trout, but no interaction between diet and stress was found following exposure to an acute stressor. After feeding, yeast diets induced higher alkaline tides in fish due to lower dietary ash content and consequently lower buffering capacity of yeast ingredients compared with fish meal. In addition, yeast diets induced haemolytic anaemia, which resulted in smaller erythrocytes and artificial elevations in MCH, especially for fish fed the WA diet. These results support previous suggestions that catabolism of high levels of nucleic acids produce reactive oxygen species that can overwhelm anti-oxidation pathways and damage erythrocytes. Overall, this study demonstrated that replacing fish meal with yeasts in rainbow trout diets reduced feed buffering capacity and induced haemolytic anaemia. Reduced buffering capacity can be beneficial for feed metabolism, but the nucleic acid content could limit the level of yeast inclusion in fish diets. Reductions in nucleic acids, specifically purine nucleotides, may mitigate anaemia effects in rainbow trout fed diets with 60 % replacement of fish meal with yeasts, though more research is needed to confirm this.
